# Advancing understanding of influences on cervical screening (non)‐participation among younger and older women: A qualitative study using the theoretical domains framework and the COM‐B model

**DOI:** 10.1111/hex.13346

**Published:** 2021-09-02

**Authors:** Bernadine O'Donovan, Therese Mooney, Ben Rimmer, Patricia Fitzpatrick, Grainne Flannelly, Lorraine Doherty, Cara Martin, John O'Leary, Mairead O'Connor, Linda Sharp

**Affiliations:** ^1^ Royal College of Surgeons in Ireland (RCSI) Dublin Ireland; ^2^ Programme Evaluation Unit, National Screening Service Dublin Ireland; ^3^ Population Health Sciences Institute, Newcastle University Centre for Cancer, Newcastle University Newcastle upon Tyne UK; ^4^ School of Public Health, Physiotherapy & Sports Science, University College Dublin Dublin Ireland; ^5^ National Maternity Hospital, St Vincent's Hospital Dublin Ireland; ^6^ National Screening Service Dublin Ireland; ^7^ Department of Histopathology Trinity College, University of Dublin Dublin Ireland; ^8^ Department of Pathology Coombe Women and Infants University Hospital Dublin Ireland; ^9^ School of Public Health University College Cork Cork Ireland

**Keywords:** barriers, cancer risk, cervical cancer, COM‐B model, screening coverage, Theoretical Domains Framework

## Abstract

**Background:**

Effective screening can prevent cervical cancer, but many women choose not to attend their screening tests.

**Objective:**

This study aimed to investigate behavioural influences on cervical screening participation using the Theoretical Domains Framework (TDF) and COM‐B models of behaviour change.

**Design:**

A qualitative study and semistructured phone interviews were conducted with women invited for routine screening tests within the national cervical screening programme in Ireland.

**Setting and Participants:**

Forty‐eight women aged 25–65 years were recruited from the national screening register.

**Results:**

Seven core themes were identified that mapped to three COM‐B components and 11 TDF domains: (1) knowledge of cervical cancer and screening, (2) coping with smear tests, (3) competing motivational processes—automatic and reflective, (4) cognitive resources, (5) role of social support, (6) environmental influences and (7) perceputal and practical influences. A range of knowledge about screening, perceived risk of cervical cancer and human papillomavirus infection was evident. Factors that influenced screening behaviours may be hierarchical—some were assigned greater importance than others. Positive screening behaviours were linked to autonomous motivation. Deficits in physical and psychological capability (inadequate coping skills) were barriers to screening, while physical and social opportunity (e.g. healthcare professional ‘champions’) could facilitate participation. Older women raised age‐related issues (e.g. screening no longer necessary) and had more negative attitudes to screening, while younger women identified practical barriers.

**Conclusions:**

This study provides insight into screening participation and will aid development of theoretically informed interventions to increase uptake.

**Patient or Public Contribution:**

Women invited for screening tests through the national screening programme were interviewed. A Public & Patient Involvement (PPI) Panel, established to provide input into all CERVIVA research projects, advised the research team on recruitment materials and were given the opportunity to review and comment on the interview topic guide. This panel is made up of six women with various cervical screening histories and experiences.

## 1 INTRODUCTION

Cervical cancer is a global public health issue with an estimated worldwide incidence of approximately 570,000 new cases in 2018, of which over 116,000 occurred in Europe.^1^ Cervical screening programmes operate in many countries and are effective in reducing the incidence of and mortality due to cervical cancer.^2^ However, data from the screening registers of 19 European states indicates that screening coverage can vary considerably: from 10% to 79%.^3^ Over the past decade, a pattern of falling uptake has been reported in several countries. Initially, there were concerns in some developed countries about uptake in younger women;^4^ however, in recent years, a new pattern of lower uptake in older women has emerged in England and Ireland (https://www.cancerresearchuk.org/health-professional/cervical-cancer-screening-and-diagnosis-statistics#heading-Five).^5^

It is important to encourage older women to attend screening as the incidence of and mortality due to cervical cancer remain high in this age group.^2^ Screening older women can reduce their cancer risk—women who are screened in their early 50s have a 75% lower risk of developing cervical cancer between 55 and 59 years.^6^ Considerable research has investigated the factors that are associated with women's screening participation. Demographic factors such as ethnicity^7^, ^8^; practical and environmental factors such as accessible appointments and female smear takers^6^, ^9^, ^10^; and psychosocial influences such as trust,^10^, ^11^ embarrassment, anxiety^7^, ^12^, ^13^ and concerns about pain/discomfort^6^, ^13^ have been identified as being related to screening participation. Age differences also exist, with younger women reporting practical barriers, embarrassment and the perception that they are at low risk of developing cancer.^9^, ^10^, ^12^, ^13^ In contrast, older women may make active decisions not to participate; in one study, older women reported low levels of worry about cervical cancer and also perceived themselves to be at low risk of developing cancer.^13^ However, the research that has generated these findings has frequently lacked a theoretical grounding.

Assessments of the strategies and interventions that have been tested to increase cervical screening uptake have found mixed evidence of increased participation.^14^, ^15^, ^16^ In part, this may be because intervention development has not always been informed by key requirements, that is, empirical data linked to an appropriate theoretical underpinning.^17^

The Theoretical Domains Framework (TDF) is a comprehensive integrated theoretical framework—synthesized from 128 theoretical constructs from 33 theories—that can guide the identification of theoretical constructs that influence behaviour.^18^, ^19^ The TDF model can be condensed into an overarching behavioural model—the COM‐B model—with three central components, capability, opportunity and motivation, that interact in behavioural processes.^18^ The TDF and COM‐B models have been used to inform intervention design in various healthcare settings,^20^, ^21^, ^22^ but have not previously been used to examine influences on cervical screening behaviours.

In the current study, we aimed to identify factors that influence women's decisions on cervical screening (non)‐participation using the TDF and COM‐B models, with a secondary objective of comparing and contrasting factors relevant for younger and older women.

## 2 METHODS

### 2.1 Design and setting

This study took place in Ireland. In‐depth semistructured telephone interviews were conducted among women who had been invited to attend for routine/follow‐up cervical screening tests. The study was carried out as a partnership between CERVIVA (the Irish Cervical Cancer Screening Consortium) and CervicalCheck, Ireland's national cervical screening programme. This programme offers free screening tests to women in Ireland aged 25–65 years. Invitation letters are issued, to women on their register, to remind them when their next screening test is due. Women can book their test at any CervicalCheck registered GP or clinic. Under human papillomavirus (HPV) primary screening protocols, women aged 25–29 years are screened every 3 years, while those aged 30–65 years are screened every 5 years. Ethical approval was obtained from the Royal College of Physicians of Ireland (RCPI RECSAF 74). While this study was being conducted, a high‐profile controversy developed in CervicalCheck when the Health Service Executive confirmed that an audit had revealed that over 200 women who developed cancer had not been told of earlier smear tests that were misdiagnosed. A scoping inquiry into issues relating to CervicalCheck and a review of the screening histories of all women who had developed cervical cancer by the Royal College of Obstetricians and Gynaecologists (RCOG) took place in 2018 and 2019, respectively.^23^, ^24^

### 2.2 Sampling and recruitment

The CervicalCheck register of women in Ireland aged 25–65 years is compiled, and maintained, using numerous data sources including the Department of Social Protection and self‐registration by women. A purposive sample of women who had been invited to attend for routine or follow‐up (i.e., following a borderline cytology result in a routine test) screening tests was selected from this register. Purposive sampling strata were as follows: (1) age group: younger (younger than 50 years) or older (50 years or older) and (2) screening history: adequate history (attended all routine screening tests that they had been invited to since 2008) or inadequate history (attended some or no screening tests that they had been invited to since 2008), resulting in four study groups. Women undergoing colposcopy clinic surveillance (for abnormal cytology), with cervical cancer or awaiting smear test results were not eligible. Data were extracted and quality‐checked by CervicalCheck staff in April 2018. CervicalCheck invited 600 women to be interviewed in two recruitment cycles. Each invitation included an information sheet, a reply slip, a consent form and a prepaid envelope. One reminder letter was sent to nonresponders. Recruitment ended when saturation was achieved.^25^

### 2.3 Interviews

A semistructured topic guide (Appendix A) was developed from literature review and informed by the TDF model. The original 12‐domain version of the TDF model (v1) was selected as a comprehensive tool that would identify a broader spectrum of potential influences on screening behaviours.^26^ Signed consent forms were returned by all participants before interviews. Interviews were conducted by a CERVIVA researcher between August and December 2019 and lasted 45–75 min (mean 60 min). Verbal consent to record the interviews was obtained, and recordings were transcribed verbatim and anonymized. A ‘One4All’ gift voucher (€25) was sent to participants once the interviews were completed to thank them for their time. All personal data were handled in accordance with the General Data Protection Regulation (GDPR), 2018.

### 2.4 Coding and analysis

Transcripts were imported into NVivo 10 and an iterative analysis was performed concurrently with data collection. A combination of inductive thematic and deductive framework analyses using the TDF model was conducted. The transcripts were reviewed through familiarization, construction of a thematic framework (TDF domains), indexing, sorting of data and interpretation.^27^ Salient text blocks referring to barriers and facilitators were inductively identified and coded. Two researchers (B. O'D., B. R.) independently read and coded 10 transcripts; recurrent themes and coding were compared and discussed, with differences resolved by consensus. Coding was finalized, and the remaining interviews were coded by one researcher (B. O'D.); in separate steps, each theme was deductively mapped to the TDF domains and then to the COM‐B model. Other members of the research team were consulted as and when needed. Analysis identified the similarities and differences across age and screening histories. Themes were deductively mapped to the three COM‐B components and their subdivisions: Capability (psychological, physical), Motivation (automative, reflective) and Opportunity (social, physical), and 12 TDF domains (*Knowledge, Skills, Social/professional role and identity, Beliefs about capabilities, Beliefs about consequences, Motivation and goals, Memory, attention and decision processes, Environmental context and resources, Social influences, Emotion, Behavioural regulation* and *Nature of the behaviours*).

Where appropriate, illustrative anonymized quotes from the study participants are included in Section Design, 3.

## 3 RESULTS

### 3.1 Characteristics of the study participants

Interviews were conducted with 48 women—34 were adequately screened (AS) and 14 women were inadequately screened (IS); 17 women were younger than 50 years of age, while 31 women were 50 years of age or older. Detailed information about the participants is shown in Table 1.

**Table 1:** Characteristics of the study participants (*n* = 48)

Demographics	Number (*n*)
Age at interview (years)	
Below 40	9
41–50	11
51–60	24
61–70	4
Screening history	
Adequate^a^	34
Inadequate^b^	14
Relationship status	
Married/cohabiting	36
Separated	5
Divorced	3
Single	4
Education	
Tertiary level^c^	22
Diploma/certificate	15
Leaving certificate^d^	11
Employment	
Employed/self‐employed	38
Retired	3
Unemployed	2
Other	5
Previous abnormal cytology result(s)	
Yes^e^^,^ f	34
No	14
Ethnicity	
White Irish	44
White English/Other	3
Mixed	1

^a^ Self‐reported had attended all routine CervicalCheck screening tests.

^b^ Attended some (*n* = 9) or no CervicalCheck screening tests (*n* = 5).

^c^ Post‐secondary education, for example, university and/or higher education institutions.

^d^ School‐leaving qualification.

^e^ Self‐reported had an abnormal smear at some time in the past, either through CervicalCheck or private cytology tests.

^f^ Of these, 30 had an adequate screening history and 4 had an inadequate screening history.

### 3.2 Summary of core themes, TDF domains and COM‐B constructs

Seven core themes were identified in relation to women's cervical screening participation: (1) Knowledge of cervical cancer and cervical screening; (2) Coping with smear tests; (3) Competing motivational processes—automatic and reflective; (4) Cognitive resources; (5) Role of social support; (6) Environmental influences; and (7) Perceptual and practical influences. Table 2 presents the subthemes and themes that emerged during analysis. Results are presented according to each of the core themes and relevant COM‐B construct and TDF domain(s) with illustrative quotes; example quotes are included in the main body of the paper. Additional quotes linked to TDF domains and COM‐B constructs are presented in Table S3 in the Supporting information.

**Table 2:** Subthemes and themes linking to relevant TDF domains and COM‐B constructs

Subthemes	Core theme	TDF domains	COM‐B constructs
Knowledge—procedural	Knowledge of cervical cancer and cervical screening	Knowledge	Psychological capability
Knowledge—cervical cancer			
Knowledge—HPV infection			
Cervical cancer risk factors			
Perception of own cervical cancer risk			
Memory lapses/aids	Cognitive resources	Memory, attention and decision processes	Psychological capability
Salient events			
Perceptual/practical barriers	Perceptual and practical influences	Behavioural regulation	Psychological capability
Priority setting			
Facilitators			
Routine/habit	Coping with smear test	Nature of the behaviours	Psychological capability
Direct experience			
Coping skills	Coping with smear test	Skills	Physical capability
Self‐efficacy			
Previous experience of smear tests	Coping with smear test	Nature of the behaviours	Physical capability
Female/experienced smear takers	Environmental influences	Environmental context and resources	Physical opportunity
HPV self‐sampling kits			
Information sources			
Social/family support	Role of social support	Social influences	Social opportunity
Group norms			
Healthcare professional ‘champions’			
Embarrassment/anxiety	Competing motivational processes—automatic	Emotion	Automatic motivation
Value of competent smear takers			
Triggering events/previous adverse experiences			
Evaluation of screening	Competing motivational processes—reflective	Beliefs about consequences	Reflective motivation
Self‐efficacy	Competing motivational processes—reflective	Beliefs about capabilities	Reflective motivation
Perceived competence			
Optimism			
Autonomous/self‐determined motivation	Competing motivational processes—reflective	Motivation and goals	Reflective motivation
Controlled /external motivation			
Lack of symptoms	Competing motivational processes—reflective	Knowledge	Reflective motivation

Abbreviations: HPV, human papillomavirus; TDF, Theoretical Domains Framework.

### 3.3 Thematic results

#### 3.3.1 Knowledge of cervical cancer and screening (COM‐B construct—psychological capability; TDF domain—knowledge)

Knowledge of cervical cancer mapped onto the COM‐B component of psychological capability and the TDF knowledge domain. Factors related to psychological capability included procedural knowledge; knowledge about cervical cancer, HPV infection and the risk factors for cervical cancer; and women's perception of their own risk of developing cervical cancer.

Most interviewees felt that they had a good level of knowledge about screening. However, some older women—both AS and IS—were confused about screening processes. This confusion centred on poor system knowledge about eligibility criteria, frequency of invitation letters and how samples are processed. These knowledge deficits also emerged in relation to knowledge about HPV—many older women demonstrated limited/no HPV knowledge compared to younger women. Poor procedural knowledge often meant that women were surprised by their invitation, questioned its timing or their eligibility and therefore did not make arrangements to attend:

I wasn't sure if it [invitation letter] was real, or what it was, [laughing] whether it was one of those things you get in the post.

(DS300025, IS, 43 years)

Many interviewees felt that they were informed about cervical cancer and regarded it as a ‘serious’ disease. On the other hand, some interviewees demonstrated poor levels of knowledge about which women are at the highest risk of developing cervical cancer:

I don't know I'm not sure if it's a certain age group that's more susceptible or not

(DS30032, AS, 42 years)

Some differences emerged; more knowledge deficits about higher risk groups were evident among IS women compared to AS women, with limited/no knowledge particularly evident among younger IS women.

Women's perceptions of their own risk of developing cervical cancer varied. AS women, regardless of their age, stated that they considered themselves above medium risk or high risk for developing cervical cancer. They linked this perception to previous abnormal results or their general concerns about all cancers. In contrast, IS women perceived themselves to be at low risk of developing cervical cancer.

#### 3.3.2 Coping with smear tests (COM‐B construct—physical capability; TDF domain—skills)

The theme of coping with smear tests mapped to the COM‐B component of physical capability and the TDF skills domain. One factor that emerged in relation to physical capability was women's intrinsic coping skills. Both IS and AS women (in both age groups) spoke about finding smear tests uncomfortable and stressful. However, AS women, both younger and older, while acknowledging the negative aspects of smear tests, were confident in their personal ability to cope and maintain their patterns of attendance. This self‐efficacy was often linked to their use of coping techniques such as deep breathing and distraction as well as their accumulated experience of screening. Many older women, both AS and IS, spoke about how they felt less embarrassment attending for screening as they aged. They linked this change in attitude to their increasing maturity, experiences of childbirth and their coping skills. In contrast, some AS older women felt increased anxiety that they linked to embarrassment about changes to their bodies as they aged:

I think seeing your body ageing, you get a bit shy about it. More shy.

(DS300011, AS, 50 years)

#### 3.3.3 Competing motivational processes—automatic processes (COM‐B construct—automatic motivation; TDF domain—emotion)

The theme of competing motivations—automatic processes maps to the COM‐B construct of automatic motivation and the TDF domain of emotion. Automatic motivation involves instinctive and affective processes that can stimulate or inhibit women's positive screening behaviours. These included inhibiting factors: women's feelings of embarrassment/anxiety about smear tests; the mediating effects that competent smear takers could have on negative emotions; and potentially ‘triggering’ effects of previous adverse experiences of smear tests or a history of sexual assault.

Many women across all four groups highlighted the embarrassment that they felt during their smear tests as well as the invasiveness of the procedure. However, many women spoke about the benefits of competent and experienced smear takers in reducing women's anxiety:

You need to have somebody that's fairly confident that can do it quite quickly, quite promptly, and that's very experienced. That probably works best with your more anxious lady.

(DS300014, AS, 53 years)

Both AS and IS women reported difficult smear tests in the past; however, IS women often described lasting anxiety/fear because of these negative experiences, for example, incompetent smear takers. They spoke about their instinctive desire to avoid smear tests:

I do remember the one [smear test] I had when [name] was born and it wouldn't have been a great experience and I kind of said to myself ‘Oh no I don't want to go back through this’

(DS3006, IS, 60 years)

Some women also highlighted that competently trained smear takers were essential as the procedure could be significantly more difficult for women who have experienced sexual assault and could trigger extreme distress/trauma.

In contrast, some AS women described screening attendance as a habitual behaviour that began with a postpartum test and was maintained:

I suppose it's [screening] routine, isn't it? I had to get one [smear test] done after [my child] was born, but even though I have been delayed sometimes in getting them and booking them, I have stayed up to date, I have got all the tests done after that I should have done

(DM550492, AS, 34 years)

#### 3.3.4 Competing motivational processes—reflective processes (COM‐B construct—reflective motivation; TDF domains—beliefs about consequences, beliefs about capabilities, motivation and goals, knowledge)

Reflective motivational processes map to the COM‐B construct of reflective motivation and the following TDF domains: beliefs about consequences, beliefs about capabilities, motivation and goals and knowledge. Reflective motivation involves conscious thought processes that can activate or discourage women's screening attendance. These processes included: evaluation of screening; autonomous/self‐determined motivation; and controlled/external motivation.

Women across the two groups (AI and IS) reported perceiving similar advantages and disadvantages to screening. The positive aspects included the following: it being a free service; providing peace of mind; being potentially lifesaving; and providing early detection of problems. Most AS women, regardless of age, acknowledged the negative aspects of the procedure (such as discomfort, intrusiveness, pain), but were more focused on the health benefits of screening. Some AS and IS women, irrespective of age, reported significant concerns about delays in getting results and the accuracy of the results:

Whereas now I'm like even if I go and have it [smear test], I could have it [cervical cancer] and they still won't find it

(DM550499, IS, 56 years)

Older and younger IS women differed in their evaluations of screening. Older women often felt that screening was no longer necessary for them, as they were older and/or were not sexually active. Despite this viewpoint, they mostly had positive perceptions of cervical screening and felt that it had significant benefits and relevance for younger women. In contrast, IS younger women described problems: the invasiveness of the procedure itself; lack of information when abnormal results were received; extended waiting times for results; and anxiety related to further testing/repeat smears:

In terms of negatives, for some people it could cause a lot of anxiety because they might need repeat smears when there's nothing wrong

(DM550498, IS, 32 years)

Generally, AS women displayed stable intentions to participate; older women in this group usually arranged their screening appointment promptly, while younger women spoke about how they were frequently delayed in making appointments. However, these delays were usually unintended and their intrinsic/self‐determined goal was to keep up to date with their screening. In contrast, IS women, both older and younger, were not similarly motivated and often spoke about having conflicting priorities. For some women, this was because they did not have concerning symptoms:

I don't feel I have any symptoms, or any pain or discharge, or any reason to have it [smear test] done.

(DS3008, IS, 62 years)

A few IS women had attended for some smear tests in the past because of external or controlled reasons. These included recent health concerns in the family or to gain the approval of family/friends. However, women who felt pressured to participate for these external reasons often did not persist with screening:

So, I did go back then four or maybe five years ago, and of course it was no problem, so it's not that there was a problem, it's just I was going on holidays whenever it came up, and then I let it lapse and I haven't gone.

(DS3006, IS, 60 years)

#### 3.3.5 Cognitive resources (COM‐B construct— psychological capability; TDF domain—memory, attention and decision processes)

In terms of cognitive resources, factors related to psychological capability (which mapped to the TDF domain of memory, attention and decision processes) included memory lapses; memory aids/prompts; and past salient events.

Most women, regardless of age or screening history, could not recall how many screening invitations they had received. They reported that they often put invitations in ‘safe places’ and then forgot about them. Reminder letters were considered useful and prompted many women across the groups to make appointments:

I'm happy to get the prompting letters…I wouldn't see it as an annoyance…We have such busy lives we have to be reminded.

(DM550494, IS, 50 years)

When cued by their invitation letter, many AS younger women considered salient events (such as past abnormal results, family history of cancer) and allocated attentional priority to screening.

#### 3.3.6 Role of social support (COM‐B construct*—*social opportunity; TDF domain—social influences)

The theme of social support maps to the COM‐B construct of social opportunity and the TDF social influences domain. Factors related to social opportunity included: social influences such as social/family support; creating group norms; and health professional ‘champions’.

Generally, women made decisions about attendance themselves and did not seek advice from others, regardless of their screening histories. Some AS women talked to their family/friends about upcoming screening tests, indicating that attending for cervical screening was an established behaviour in their social group that could be discussed without embarrassment.

There was a pattern of advocacy among the older women; many AS and some IS women encouraged their family/friends/work colleagues to participate in screening. Some older IS women had recently decided to participate in screening when personally encouraged by their health professionals to attend. These health professional ‘champions’ had engaged with the individual woman about screening during a routine medical appointment:

It was really the nurse in the practice who… I think I was getting stitches out or something, or having some test or other done, and it was she who highlighted it and she got me put on the register.

(DM550496, IS, 57 years)

In contrast, younger IS women reported that family support had been a critical factor in their recent decisions to attend. This support often originated from partners who had read the CervicalCheck information sheets and urged the women to prioritize their health and find time to attend.

#### 3.3.7 Environmental influences (COM‐B construct—physical opportunity; TDF domain—environmental context and resources)

Environmental influences mapped to the COM‐B construct of physical opportunity and the environmental context and resources TDF domain. The factors that linked to physical opportunity included: smear takers; HPV self‐sampling kits; and use of information sources.

Irrespective of age or screening histories, women expressed their preference for female and/or experienced smear takers. Some younger IS women described problems with their local smear takers that inhibited their attendance, but they had not made any plans to access different GPs:

If there were a clinic or a different patient section in a hospital or whatever, I would have no problems, but it was just the nursing staff at my GP's that I don't like

(DM550495, IS, 41 years)

Most of the IS women, younger and older, reported that they would use HPV self‐sampling kits if they received one in the post. They felt that kits would be useful and more convenient for them, but highlighted the importance of clear, ‘step‐by‐step’ instructions. In contrast, many AS older women said that they would not as they lacked confidence that they would do it correctly.

Similar information sources—such as healthcare professionals, family/friends and the internet—were available to most of the women. AS women spoke about using these resources in the past—often prompted by their CervicalCheck invitations—to find out about cervical cancer and/or screening. IS women did not generally research these topics, but indicated that they would use similar sources if such information was required. GPs were reported as the most trusted source of health information, but younger women—both AS and IS—often accessed multiple information sources:

It wouldn't be just one step. I wouldn't take the doctor's word for it; I would want my own research done. Because not one person knows everything. You can't say that everything on the internet is true.

(DM550495, IS, 41 years)

While specific information sources were not linked to screening participation, several women who were IS highlighted the merits of health websites with supportive components such as patient forums and webchats.

#### 3.3.8 Perceptual and practical influences (COM‐B construct—psychological capability; TDF domain—behavioural regulation)

Perceptual and practical influences mapped to the COM‐B construct of psychological capability and the TDF behavioural regulation domain. Women's ability to regulate screening behaviours often linked to perceptual and practical factors—both inhibitory and stimulating—and their prioritization of screening.

There were distinct age differences in reported barriers to screening. Many older IS women felt that screening was no longer necessary because of their age/lifestyle:

A few years ago, when I was younger, I did attend some of them. But. With my age…and I'm not sexually active. I just didn't think it was necessary

(DS30008, IS, 62 years)

This contrasted with the health beliefs of many older AS women who felt that screening was an essential part of their healthcare, with benefits that outweigh the inconvenience.

In contrast, most younger women—both AS and IS—highlighted practical barriers to screening such as time pressures, difficulties with childcare and accessing appointments. Many younger women also considered screening a low priority in their lives because as mothers, they often had competing demands on their time, were not worried about cervical cancer, were in a monogamous relationship or displayed no symptoms.

Although AS women felt that screening was necessary for maintaining good health, many AS older women described age‐related barriers—increasing difficulties with procedure as they got older, due to postmenopausal changes—psychological (e.g., increased anxiety) or physical (e.g., vaginal dryness):

I just hate getting them done. I find that, maybe as I'm getting older, I don't know, I find it very uncomfortable and very painful. Maybe because I am a bit tense, or something, about it as well. I just find it, and I know it has to be done, and so forth, but I just found the last couple of ones were very uncomfortable

(DS30016, AS, 55 years)

Both AS and IS women described factors that could potentially encourage screening attendance such as flexible screening appointments/access to GP practices or practical tips such as pelvic tilts during the procedure or taking painkillers before the smear test.

## 4 DISCUSSION

This is the first study to use the combination of TDF and COM‐B models to identify influences on cervical screening behaviours. We identified seven themes: (1) knowledge of cervical cancer and cervical screening; (2) coping with smear tests; (3) competing motivational processes—automatic and reflective processes; (4) cognitive resources; (5) environmental influences; (6) role of social support; and (7) perceptual and practical influences. These were linked to six COM‐B elements and 11 TDF domains. The COM‐B and TDF models provide a framework to systematically identify the influences on screening behaviours and, hence, targets for strategies to increase screening participation. The use of the combination of the COM‐B and TDF models had additional benefits helping to highlight the underlying influences that both inhibit and promote screening. This links from the TDF domains to the associated Behavioural Change Wheel (BCW),^19^ meaning that relevant intervention functions and supporting policies that could facilitate behaviour change/increase screening attendance can be identified. Moreover, further links to the Behavioural Change Taxonomy^28^ can provide suggestions as to possible behavioural change techniques for consideration in intervention development. This study therefore provides a direct springboard for the development of evidence‐based and theoretically informed interventions to improve screening uptake.

Consistent with previous research,^12^, ^13^, ^29^ many IS women reported limited knowledge about cervical cancer risk factors and believed that they were at low risk of developing it. In contrast, many older women who considered screening unnecessary because of their age or current sexual activity positively assessed cervical screening. These findings suggest that a hierarchy of influences could exist in determining screening behaviours among older women—the perceived personal relevance of screening was more important than their knowledge of its overall health benefits. Improving knowledge of cervical cancer and screening alone is likely to be insufficient for effective behavioural change strategies.^30^ This suggests that modelling and environmental restructuring interventions that increase psychological capability would be useful. Strategies tailored for older women that target their beliefs about salience of screening, which can change over time as they age, would be worth testing. Relevant advertising campaigns with high‐profile ‘older’ women modelling positive screening behaviours or on‐screen prompts for GPs to ask older women about screening could also prove effective.

Women highlighted the invasiveness of undergoing a screening test and, as with other studies, described high levels of stress and anxiety before their appointments.^12^, ^13^ AS women were confident in their abilities to cope with negative aspects of screening such as finding the test intrusive. Coping strategies such as breathing techniques were commonly used, suggesting that prescreening information that offered women tips and advice on such coping strategies could be useful. As with previous research, many older women reported increasing problems with the smear test procedure—linked to menopausal changes—as they aged.^13^ These findings highlight the necessity of supporting older AS women and building physical capability to maintain their adherence. This can be achieved with physical skill development through training or enabling interventions.^20^ Practical advice on lubrication or pain medication in information leaflets or as part of standard verbal instructions from smear takers could increase individuals’ skills and be beneficial for these older women. The current study also found that promoting physical opportunity with HPV self‐sampling kits could prove effective as older women who were IS indicated that they would use HPV kits if given the opportunity to do so. Such kits could be enabling interventions, provided they were accompancied with additional support to develop women's confidence. This additional support could include online video tutorials of self‐sampling; information sheets with step‐by‐step instructions (both clear explanatory text and a visual guide); or ‘practise’ sessions across healthcare settings, where women are supported by experienced smear takers, receiving guidance from HCPs during their self‐collection/while using the kits themselves.

A key finding of this study was that both motivational processes, automatic and reflective, were important in influencing screening behaviours. In contrast to IS women, those who were AS were autonomously motivated to attend screening—salient events such as previous abnormal results played a role in their prioritization of screening. The self‐determination theory (SDT) on health behaviours suggests that intrinsic motivation will increase when psychological needs of autonomy, competence and relatedness are satisfied.^31^, ^32^ Potential strategies to increase intrinsic motivation, which support autonomy, relatedness and encourage identification with the value of screening, could include motivational interviewing, peer support and relevant information with meaningful rationales for behaviour change.^32^, ^33^ These approaches would augment automatic motivation and could prove beneficial in increasing uptake in IS women. Application of the BCW suggests that changes to service provision that focus on training and enablement interventions could help establish and maintain screening attendance over time.

Social support was a significant influence, further highlighting the role of relatedness in reinforcing women's attendance by creating group norms of positive screening behaviours within families/friendship groups.^32^, ^33^, ^34^ It should be noted that the CervicalCheck controversy was part of the social environmental context at this time. The high‐profile events that were occurring may have impacted on women's views on screening and our findings. However, additional aspects of social support emerged in this study among IS women. Younger women in this group identified the impact of family support in encouraging them to attend, while IS older women reported the positive effects of a personal interaction with their health professionals on their screening behaviours. While previous research has identified the central role of healthcare providers in supporting screening,^35^, ^36^, ^37^ the current study offers information specific to older women. The theory of planned behaviour model identifies a gap between intention and behaviour.^38^ Our findings suggest that family support may act as a moderating factor on this gap for younger IS women, while health professional ‘champions’ who encourage women to attend screening may possibly have a similar moderating effect for older women who are IS. However, it should be noted that controlled motivation may not be a reliable influence on screening behaviours over time.^39^ SDT suggests that women who are motivated to attend screening for controlled reasons—perceived approval of others—feel a sense of obligation and will only persist if the external pressure is maintained.^33^, ^39^ Our findings suggest that persuasion and education interventions that link to environmental/social planning and communication/marketing policies could prove effective in maximizing the positive effects of social opportunity on screening behaviours.

Psychological capability was found to be influential in screening behaviours; as with previous research, practical barriers were commonly reported by younger women (for example, lack of time, arranging childcare and time off work), while older women cited issues such as perceived irrelevance of screening for them and concerns associated with postmenopausal changes/ageing bodies.^8^, ^13^ Psychological capability can be augmented with modelling and environmental restructuring interventions.^20^ This suggests that system changes that increase convenience and provide prompts such as drop‐in clinics, evening/weekend appointments and text reminders could improve uptake in younger women. Different approaches are required for older women such as targeted information campaigns with relevant data such as latency periods from HPV exposure, high‐risk groups and the possibility of HPV reactivation around menopause. In this context, policy changes in communication/marketing with campaigns across multiple media platforms and guidelines (such as establishing age‐specific HPV infection communication documents within the screening programme) would also be valuable.

### 4.1 Strengths and limitations

A major strength of the current study is the use of the COM‐B and TDF models in examining influences on screening behaviours. The COM‐B and TDF models provided a framework to explore and identify the complex factors that influence these behaviours. A comprehensive behavioural analysis of influential factors could be undertaken, which is an essential step in developing theoretically informed interventions.^40^ The links between the TDF and COM‐B models to the BCW provide a systematic process to fully understand the nature of screening participation, characterize interventions and link these to specific policy categories. The TDF model also informed the topic guide, which enabled information to be gathered on women's typical screening behaviours. Another strength is the inclusion of women with a range of screening histories. This proved insight into an understudied cohort—those who do not (consistently) engage with screening. We also distinguished younger and older women, which is important, given the indications here of how influences on behaviour may differ in these groups. However, as in any qualitative study, women who were interviewed (both AS and IS) were motivated to participate and may have distinctive views/opinions about screening. Moreover, it is worth remembering that the interviews took place against the background of a high‐profile controversy around CervicalCheck. It is possible that this may have impacted on women's views on screening and, hence, on our findings.

## 5 CONCLUSION

This study has identified that women's screening decisions were influenced by a variety of factors, some of which can evolve over time. Establishing positive screening behaviours that persist will require tailored strategies that support autonomous motivational processes and increase capability (physical and psychological) and opportunity (physical and social). The study findings can be mapped to specific intervention functions, thereby taking a step towards the development of evidence‐based and theoretically informed interventions to improve screening uptake.

## ACKNOWLEDGEMENTS

The authors would like to thank the women who agreed to be interviewed for this study. We would also like to acknowledge the technical assistance of CervicalCheck IT services. This study was funded by the Health Research Board Applied Partnership Awards (APA‐2016‐1874) and the National Screening Services. Open access funding provided by IReL. WOA Institution: Royal College of Surgeons in Ireland Blended DEAL: IReL.

## CONFLICT OF INTERESTS

The authors declare that there are no conflicts of interest.

## AUTHOR CONTRIBUTIONS

M. O'Connor, C. Martin and L. Sharp conceived and designed the study. M. O'Connor, L. Sharp, C. Martin, T. Mooney, P. Fitzpatrick, G. Flannelly and B. O'Donovan developed the study methods. M. O'Connor, T. Mooney, L. Doherty and B. O'Donovan assisted in acquiring data. C. Martin and M. O'Connor managed the project. B. Rimmer, L. Sharp, M. O'Connor and B. O'Donovan contributed to the analysis and interpretation of data; all authors critically reviewed the manuscript and approved the final version.

## Supporting information

Supporting information.

## Data Availability

Data are available on request from the corresponding author, but not publicly available due to privacy or ethical restrictions.

## APPENDIX A TOPIC GUIDE

### Introduction

‘Hello…it's XX from xx, we spoke last week and arranged to talk about your views on smear tests and cervical screening. Is it still a good time for you?’

Verbal consent

‘Just to remind you…’

Start recording

‘I'll start the tape now…’

Assure of confidentiality

‘Please be assured that everything you say’.

Summarize purpose and structure again

‘Before we start I'ld just like to tell you a bit about the study. We are exploring womens' views of smear tests and cervical screening and what influences their decisions to attend for screening tests. We want to hear the views of a variety of women—so women who attend regularily, those who attend sometimes and those who never attend. We also want to find out what women think would improve screening/smear tests’.

'So how does that sound? Do you have any questions before we begin?'

### Background (demographic information)

‘So to start off can you tell me a bit about yourself?' (raised questioning tone & pause)

• Cervical screening history

‘Have you ever received an invitation to take part in CervicalCheck?'

**If not**

‘Has your doctor/nurse at your GPs surgery ever mentioned having smear tests?'

‘What were your first thoughts when you got your first invitation letter from CervicalCheck?'

‘Did these thoughts/your thinking change when you got your second invitation?…Did you make arrangements to have a smear test?…Was it long after getting your invitation?'

‘Why did you decide to have your screening test?'

‘What did you consider when you were making your decision?'

‘Did you talk to anyone about it?'

**If not**

‘Why did you decide not to have your CervicalCheck screening test?'

‘What did you consider when you were making your decision?'

‘Did you talk to anyone about it?'

‘Did you know about/were you aware of cervical screening before you got the CervicalCheck invitations?'

‘Have you participated in other screening programmes e.g. BreastCheck, Diabetic RetinsScreen?…Can you tell me about that?'

### Undergoing the screening test

‘So just to return to your most recent screening test. Can you tell me about it?'

‘What do you think would make undergoing the screening test easier?'

‘Was there anything particularily difficult about undergoing the test?' [Physical/Psychological elements]

‘What might make you more likely to participate in screening?…There is a screening tool that you could use yourself at home called HPV self‐sampling. Have you heard of it?'

**If not**

‘So I know you decided not to have your CervicalCheck smear test but I'ld like to ask you some general questions about screening. What do you think would make undergoing the screening test easier?'

‘What might make you more likely to participate in screening?…There is a screening tool that you could use yourself at home called HPV self‐sampling. Have you heard of it?'

- Information‐seeking and social support
  ‘If you were looking for trustworthy/reliable information on cervical screening what would you do?…Would you use any other sources of information e.g. friends/medical book/Internet?'
  - HPV
  ‘Have you heard about HPV?'
  ‘What can you tell me about it?'
  ‘Have you heard of HPV testing/a HPV test?'
  'CervicalCheck may soon be changing their screening test from the current test, which looks for abnormal cells in the cervix to a test that looks for the presence or absence of HPV infection. Physically, it will feel the same as having smear tests. The changes, if implemented, will mean that all women (aged 25–60 years) will attend for screening every 5 years. This will be different from current screening protocols, where women aged 24–44 years attend for screening every 3 years.'
  ‘Do you have any thoughts on the proposed changes?'
  ‘Do you think you will be more or less likely to participate in cervical screening in the future if these changes are made?'
  - Future improvements to cervical screening
  ‘Looking back what would you have liked to know before you went for your smear test?'
  ‘What would you want a family member/friend who was invited for a smear test to know?'
  ‘What would you want a family member/friend who was going for a smear test to know beforehand?'
  ‘What would you want her to know afterwards?…How do you think she should get this information?'
  ‘Any suggestions on how the screening test experience could be improved for other women?'
  **If not**
  ‘So I know you decided not to have your CervicalCheck smear test but I'd like to ask you some general questions about improving screening…Looking back to when you were invited for a smear test (pause) was there anything/any information you would have liked to know?'
  ‘What would you want a family member/friend who was invited for a smear test to know?'
  ‘What would you want a family member/friend who was going for a smear test to know beforehand?'
  ‘What would you want her to know afterwards?…How do you think she should get this information?'
  ‘Any suggestions on how the screening test experience could be improved for other women?'
  - Close

‘Anything else you would like to tell me about cervical screening?'

Thank interviewee. Reassure again about confidentiality and repeat information provided at the beginning. Tell participant what to do if they have any questions/a list of further information can be emailed or posted to her if she requires.

## References

[1] World Health Organization Cervix uteri fact sheet. 2018. http://gco.iarc.fr/today/data/factsheets/cancers/23-Cervix-uteri-fact-sheet.pdf. Accessed January 6, 2021.

[2] Anttila A , von Karsa L , Aasmaa A , et al. Cervical cancer screening policies and coverage in Europe. Eur J Cancer. 2009;45(15):2649‐2658. 10.1016/j.ejca.2009.07.020 19699081

[3] Cancer screening in the European Union. 2017. https://ec.europa.eu/health/sites/health/files/major_chronic_diseases/docs/2017_cancerscreening_2ndreportimplementation_en.pdf. Accessed January 6, 2021.

[4] Lancucki L , Fender M , Koukari A , et al. A fall‐off in cervical screening coverage of younger women in developed countries. J Med Screen. 2010;17(2):91‐96. 10.1258/jms.2010.010017 20660438

[5] CervicalCheck Programme Report. 2016‐2017. http://www.screeningservice.ie/publications/CervicalCheck-Programme-Report-2016-2017.pdf. Accessed January 6, 2021.

[6] Cervical Cancer Trends Report. 2017. https://www.ncri.ie/sites/ncri/files/pubs/CervicalCaTrendsReport_MP.pdf. Accessed January 6, 2021.

[7] Castanon A , Landy R , Cuzick J , Sasieni P . Cervical screening at age 50‐64 years and the risk of cervical cancer at age 65 years and older: population based case control study. PLoS Med. 2014;11(1):e1001585. 10.1371/journal.pmed.1001585 24453946 PMC3891624

[8] Malagón T , Kulasingam S , Mayrand MH , et al. Age at last screening and remaining lifetime risk of cervical cancer in older, unvaccinated, HPV‐negative women: a modelling study [published correction appears in Lancet Oncol. 2019 Jan;20(1):e10]. Lancet Oncol. 2018;19(12):1569‐1578. 10.1016/S1470-2045(18)30536-9 30392810

[9] Sasieni P , Castanon A , Cuzick J . Effectiveness of cervical screening with age: population based case control study of prospectively recorded data. BMJ. 2009;339:b2968. 19638651 10.1136/bmj.b2968 PMC2718082

[10] Waller J , Bartoszek M , Marlow L , Wardle J . Barriers to cervical cancer screening attendance in England: a population‐based survey. J Med Screen. 2009;16(4):199‐204. 10.1258/jms.2009.009073 20054095

[11] Eaker S , Adami HO , Sparén P . Attitudes to screening for cervical cancer: a population‐based study in Sweden. Cancer Causes Control. 2001;12(6):519‐528. 10.1023/a:1011233007132 11519760

[12] Hope KA , Moss E , Redman CWE , Sherman SM . Psycho‐social influences upon older women's decision to attend cervical screening: a review of current evidence. Prev Med. 2017;101:60‐66. 10.1016/j.ypmed.2017.05.002 28502577

[13] Waller J , Jackowska M , Marlow L , Wardle J . Exploring age differences in reasons for nonattendance for cervical screening: a qualitative study. BJOG. 2011;119:26‐32. 10.1111/j.1471-0528.2011.03030.x 21668764

[14] Marlow L , McBride E , Varnes L , Waller J . Barriers to cervical screening among older women from hard‐to‐reach groups: a qualitative study in England. BMC Womens Health. 2019;19(1):38. 10.1186/s12905-019-0736-z 30808349 PMC6390581

[15] Everett T , Bryant A , Griffin MF , Martin‐Hirsch PP , Forbes CA , Jepson RG . Interventions targeted at women to encourage the uptake of cervical screening. Cochrane Database Syst Rev. 2011;2011(5):CD002834. 10.1002/14651858.CD002834.pub2 PMC4163962 21563135

[16] Forbes C , Jepson R , Martin‐Hirsch P . Interventions targeted at women to encourage the uptake of cervical screening. Cochrane Database Syst Rev. 2002;3(3):CD002834. 10.1002/14651858.CD002834 12137660

[17] Musa J , Achenbach CJ , O'Dwyer LC , et al. Effect of cervical cancer education and provider recommendation for screening on screening rates: a systematic review and meta‐analysis. PLoS One. 2017;12(9):e0183924. 10.1371/journal.pone.0183924 28873092 PMC5584806

[18] O'Cathain A , Croot L , Duncan E , et al. Guidance on how to develop complex interventions to improve health and healthcare. BMJ Open. 2019;9:e029954. 10.1136/bmjopen-2019-029954 PMC6701588 31420394

[19] Michie S , van Stralen MM , West R . The behaviour change wheel: a new method for characterising and designing behaviour change interventions. Implement Sci. 2011;6:42. 10.1186/1748-5908-6-42 21513547 PMC3096582

[20] Michie S , Johnston M , Francis J , Hardeman W , Eccles M . From theory to intervention: mapping theoretically derived behavioural determinants to behaviour change techniques. Appl Psychol. 2008;57:660‐680. 10.1111/j.1464-0597.2008.00341.x

[21] Graham‐Rowe E , Lorencatto F , Lawrenson JG , et al. Barriers and enablers to diabetic retinopathy screening attendance: protocol for a systematic review. Syst Rev. 2016;5(1):134. 10.1186/s13643-016-0309-2 27515938 PMC4981960

[22] Cowdell F , Dyson J . How is the theoretical domains framework applied to developing health behaviour interventions? A systematic search and narrative synthesis. BMC Public Health. 2019;19:1180. 10.1186/s12889-019-7442-5 31455327 PMC6712870

[23] Scally G Scoping Inquiry into the CervicalCheck Screening Programme. 2018. https://assets.gov.ie/9785/9134120f5b2c441c81eeed06808351c7.pdf. Accessed January 6, 2021.

[24] RCOG Independent Expert Panel . Cervical screening in cases of cervical cancer in Ireland between 2008‐2018. 2019. https://www.gov.ie/en/publication/a4e567-expert-panel-review-of-cervical-screening/. Accessed January 6, 2021.

[25] Flannery C , McHugh S , Anaba AE , et al. Enablers and barriers to physical activity in overweight and obese pregnant women: an analysis informed by the theoretical domains framework and COM‐B model. BMC Pregnancy Childbirth. 2018;18:178. 10.1186/s12884-018-1816-z 29783933 PMC5963099

[26] Ritchie J , Lewis J , McNaughton Nicholls C , Ormston R . Qualitative Research Practice: A Guide for Social Science Students & Researchers. Sage Publications Inc; 2014.

[27] Mosavianpour M , Sarmast H , Kissoon N , Collet J . Theoretical domains framework to assess barriers to change for planning health care quality interventions: a systematic literature review. J Multidiscip Healthc. 2016;9:303‐310. http://10.2147/JMDH.S107796 27499628 10.2147/JMDH.S107796 PMC4959766

[28] Michie S , Richardson M , Johnston M , et al. The behavior change technique taxonomy (v1) of 93 hierarchically clustered techniques: building an international consensus for the reporting of behavior change interventions. Ann Behav Med. 2013;46(1):81‐95. 10.1007/s12160-013-9486-6 23512568

[29] Marlow LAV , Ryan M , Waller J . Increasing the perceived relevance of cervical screening in older women who do not plan to attend screening. Sex Transm Infect. 2020;96:20‐25. 31395750 10.1136/sextrans-2019-054120 PMC7029243

[30] Braun V , Clarke V . Thematic analysis. In: Cooper H , Camic PM , Long DL , Panter AT , Rindskopf D , Sher KJ , eds. APA Handbooks in Psychology®. APA Handbook of Research Methods in Psychology, Vol. 2. Research Designs: Quantitative, Qualitative, Neuropsychological, and Biological. American Psychological Association; 2012:57‐71. 10.1037/13620-004

[31] Ryan RM , Deci EL . Self‐determination Theory: Basic Psychological Needs in Motivation, Development and Wellness. The Guildford Press; 2017.

[32] Ryan RM , Deci EL . The "What" and "Why" of goal pursuits: human needs and the self‐determination of behavior. Psychol Inq. 2000;11(4):227‐268. 10.1207/S15327965PLI1104_01

[33] Kelly MP , Barker M . Why is changing health‐related behaviour so difficult*?* Public Health. 2016;136:109‐116. 10.1016/j.puhe.2016.03.030 27184821 PMC4931896

[34] Sieverding M , Matterne U . What role do social norms play in the context of men's cancer screening intention and behavior? Application of an extended theory of planned behavior. Health Psychol. 2010;29(1):72‐81. 10.1037/a0016941 20063938

[35] World Health Organisation European Conference on Screening. 2020. https://www.euro.who.int/en/health-topics/Health-systems/pages/news/news/2020/2/nurses-administer-most-cervical-cancer-screening-in-england. Accessed January 6, 2021.

[36] O'Donovan J , O'Donovan C , Nagraj S . The role of community health workers in cervical cancer screening in low‐income and middle‐income countries: a systematic scoping review of the literature. BMJ Glob Health. 2019;4:e001452. 10.1136/bmjgh-2019-001452 PMC6528769 31179040

[37] O'Connor M , Murphy J , Martin C , O'Leary J , Sharp L , Irish Cervical Screening Consortium (CERVIVA) . Motivators for women to attend cervical screening: the influential role of GPs. Fam Pract. 2014;31(4):475‐482. 10.1093/fampra/cmu029. Epub June 12, 2014. 24927724

[38] Ajzen I . The theory of planned behaviour. Organ Behav Hum Decis Process. 1991;50(2):179‐211.

[39] Hagger MS , Hardcastle SJ , Chater A , Mallett C , Pal S , Chatzisarantis NLD . Autonomous and controlled motivational regulations for multiple health‐related behaviors: between‐ and within‐participants analyses. Health Psychol Behav Med. 2014;2(1):565‐601. 10.1080/21642850.2014.912945 25750803 PMC4346087

[40] Atkins L , Francis J , Islam R , et al. A guide to using the Theoretical Domains Framework of behaviour change to investigate implementation problems. Implement Sci. 2017;12(77):77. 10.1186/s13012-017-0605-9 28637486 PMC5480145

