# Supplementary material for: Advancing understanding of influences on cervical screening (non)‐participation among younger and older women: A qualitative study using the theoretical domains framework and the COM‐B model
Source: Health Expect. 2021 Sep 2;24(6):2023–35. doi: 10.1111/hex.13346 (PMC8628586; doi:10.1111/hex.13346)
Supplement: Supplementary file 1 — Supporting information. [file HEX-24-2023-s001.docx]

*Table 3: TDF and COM-B analysis: sub-themes, themes & illustrative quotes*

| ***Themes and related sub-themes*** | ***TDF domains*** | ***COM-B*** | ***Illustrative quotes*** |
| --- | --- | --- | --- |
| *Wide range of knowledge of cervical cancer & screening*  *Desire for more procedural knowledge (e.g. sample route)*  *Poor knowledge of high-risk groups*  *Mixed perceptions of own risk*  *Mixed knowledge about HPV/HPV testing* | *Knowledge* | *Psychological Capability* | *“But maybe more clarity on what happens to the test – where does it go…More information on what happens to the sample.”*  *(DS300027, AS, 50 yrs)*  *“I don’t know about age, whether that is relevant. I have no idea…Maybe women who have had babies, I don’t know.”*  *(DS300025, IS, 43 yrs)*  *“I would put myself as low because hereditary-wise it is not directly in my family” (DS300029, AS, 53 yrs)*  *“I don’t think people understand it at all. I don’t think they have any idea about HPV”*  *(DS300013, IS, 59 yrs)*  *“I probably have [heard about HPV] a little bit. I’m not too sure. I haven’t got very much information or much knowledge on that.”*  *(DS300019, AS, 60 yrs)* |
| *Memory aids*  *Decision making*  *Salience* | *Memory, attention & decision processes* | *Psychological Capability* | *“I put it behind the clock, I said ‘I’ll fill that out now’ and forgot about it.”” (DS300038, AS, 55 yrs)*  *“I worry about all cancers, cervical cancer more than others probably because of family background”*  *(DM550487, AS, 31 yrs)* |
| *Perceptual barriers*  *Practical barriers*  *Not a priority*  *Facilitators* | *Behavioural regulation* | *Psychological Capability* | *“At the moment, it’s just not on my priority list”*  *(DM550495, IS, 41 yrs)*  *“it was great, I was able to do it [private smear] locally as well”*  *(DM550494, IS, 50 yrs)*  *“I normally take two Nurofen, to take the edge off the uncomfortable aspect of it.”*  *(DS300034, AS, 51 yrs)* |
| *Routine/habit*  *Direct experience* | *Nature of the behaviours* | *Psychological Capability* | *“I suppose after seven kids, you know, I’m sort of used to... I’m not any more embarrassed now I’m more mature I suppose. It’s another job to do.*  *(DM550499, IS, 56 yrs)*  *“I do remember the one I had when [name] was born and it wouldn’t have been a great experience and I kind of said to myself ‘Oh no I don’t want to go back through this’”*  *(DS3006, IS, 60 yrs)* |
| *Skills*  *Self-efficacy*  *Coping techniques* *– distraction; breathing exercises* | *Skills* | *Physical Capability* | *“I suppose after seven kids, you know, I’m sort of used to... I’m not any more embarrassed now I’m more mature I suppose. It’s another job to do.*  *(DM550499, IS, 56 yrs)*  *“Once you get older and you’ve done it a few times, you just go, get it done, over with, here we go, and go on with the day”*  *(DM550489, AS, 32 yrs)*  *“Breathing..yes…relaxation exercises beforehand”*  *(DS300024, AS, 61 yrs)* |
| *Anxiety*  *Embarrassment*  *Competent/trained smear takers essential*  *Triggering for some*  *Past difficult smear tests* | *Emotion* | *Automatic Motivation* | *“I absolutely hate them..I think most women do”*  *(DS300037, AS, 53 yrs)*  *“I think fear of pain and embarrassment, it probably is a bigger block than maybe people do [sic] realise”*  *(DM550490, AS, 54 yrs)*  *“I do think it would be nice for someone who is doing a procedure, that they have been trained, and that they have a lot of practice doing it, and practice doing it with different categories, and different age groups…not just from a physical point of view, but from the emotional, psychological point of view”*  *(DS30008, IS, 62 yrs)*  *“lots of people may have some negative experiences about smears, because it may just bring flashbacks and other things, so it’s just being mindful of maybe somebody’s history and maybe what’s triggering this anxiety. They may have been the subject of a sexual assault, or early childhood assault or abuse, so it’s just staff maybe being aware of that”*  *(DS300014, AS, 53 yrs)*  *“The first one I found very difficult and it has been difficult ever since…I am dreading the next one”*  *(DS300013, IS, 59 yrs)* |
| *Evaluation of screening*   - *advantages e.g. life saving, free, ‘peace of mind’* - *disadvantages e.g. invasive, concerns about accuracy, false positives, inconvenience*   *Perceptions of screening*   - *Long term health benefits* - *not relevant (older women)* | *Beliefs about consequences* | *Reflective Motivation* | *“I would say it is mostly positive. It gives you peace of mind to a great degree.”*  *(DS300019, AS, 60 yrs)*  *“Well, the positive is if you have anything wrong, it’ll be caught in time, if you go when you’re supposed to go and not be putting it off. Yes, and then you can be treated, and the early treatment is the best for any cancer”*  *(DS30003, AS, 57 yrs)*  *“it’s a bit like going to the dentist, I don’t really want to but it’s one of those things that I think it’s just worth the momentary discomfort”*  *(DS30007, AS, 57 yrs)*  *“It’s just a concern that the accuracy of this testing and then, even when I get it done, it’s not, from my understanding, 100% that everything is fine anyway”*  *(DS30008, IS, 62 yrs)*  *“Obviously the waiting time for results and the accuracy of results”*  *(DM550488, AS, 57 yrs)*  *“At my age now I think I am okay, being honest. I am a person with one partner and I reckon that helps the situation…No. It would be more for younger people.”*  *(DS300013, IS, 59 yrs)* |
| *Self-efficacy*  *Perceived competence*  *Optimism* | *Beliefs about capabilities* | *Reflective Motivation* | *“It is not a day at the spa! I just get on with it. It doesn’t bother me”*  *(DS300029, AS, 53 yrs)*  *“It is what it is and for the sake of perhaps preventing something that’s going to kill you, you just have to put up with it don’t you?”*  *(DM550487, AS, 31 yrs)*  *“It was, as always, extraordinarily unpleasant and intrusive, and unfortunately, a necessary part of being healthy”*  *(DS300018, AS, 57 yrs)* |
| *Stability of intention to adhere*  *Not a priority*  *Intrinsic motivation*  *Extrinsic motivation* | *Motivation & goals* | *Reflective Motivation* | *“even though I have been delayed sometimes in getting them and booking them [appointments], I have stayed up to date, I have got all the tests done that I should have done.*  *(DM550492, AS, 34 yrs)*  *“it'll still end up always at the bottom of my list of things to do and I’d always find something else to do first.”*  *(DM550498, IS, 32 yrs)*  *“If it [screening invitation] was my husband, I’d have him shoved out the door…but for myself, I just didn’t.*  *(DS30006, IS, 60 yrs)*  *“So, I did go back then four or maybe five years ago, and of course it was no problem, so it’s not that there was a problem, it’s just I was going on holidays whenever it came up, and then I let it lapse and I haven’t gone.”*  *(DS3006, IS, 60 yrs)*  *“And then my sister also recently had a bit of a scare... And that was really in the back of my mind as well..I just went and I did it [smear test].”*  *(DM550494, IS, 50 yrs)* |
| *Social support*  *Group norms*  *Role of HCPs as ‘champions’ (older)*  *Family support (younger)* | *Social influences* | *Social Opportunity* | *“I would mention it to friends, yes, that I was having it done.”*  *(DS30005, AS, 38 yrs)*  *“And I suppose she [mother] would check up on me as well; she’d be like, “Oh, did you get it done now? Off you go, don’t forget.””*  *(DM550492, AS, 34 yrs)*  *“I actually would encourage anybody, any of my female friends that show any reluctance to go I’m like, no, go. I just think it’s a great service.”*  *(DS30001, AS, 51 yrs)*  *“I have encouraged my daughters, because they are so much younger. I think being a younger woman…My concerns, at my age, of any risks, their situation would be different”*  *(DS30008, IS, 62 yrs)*  *“To be honest, my husband, if it wasn’t for him, I would have never gone to get them [smear tests]. Yes, he was at me and at me to get them. He looked into it more than I did, really…He paid more attention, I think, to the literature and stuff. He was like, ‘I think it’s something that you need to do.’*  *(DS300025, IS, 43 yrs)* |
| *Role of smear taker*  *– gender; familiarity; perceived experience/competence*  *HPV self-sampling kits*  *Information resources* | *Environmental context &*  *resources* | *Physical Opportunity* | *“I liked that the nurse did it, because she was a little bit more… [laughing] She wasn’t as quick to get you out of the office. She was friendlier, and she understood. I felt more at ease with her”*  *(DS300025, IS, 43 yrs)*  *It was the practice nurse did it for me, she’s very nice. I know the lady myself, she’s a local lady, so I know her and it was fine”*  *(DM550494, IS, 50 yrs)*  *“you need to have somebody that’s fairly confident that can do it quite quickly, quite promptly, and that’s very experienced. That probably works best with your more anxious lady.”*  *(DS300014, AS, 53 yrs)*  *“So I went to the practice nurse, and it wasn't a pleasant experience. She's very nice, but she's very clumsy..she really made me livid. I was very uncomfortable and very sore.”*  *(DM550496, IS, 57 yrs)*  *“I would be thinking, have I done it right? It is uncomfortable to be poking around there. Would it be done correctly?”*  *(DS300031, AS, 51 yrs)* |
| *Barriers & facilitators*  *Perceptual/practical difficulties*  *Body difficulties (age related/ postmenopausal issues)*  *Priorities* | *Behavioural regulation* | *Physical Opportunity* | *“The fact that you have to go into hospital, prepare for a day you are out of work”*  *(DS30013, IS, 59 yrs)*  *“there’s a lady here who mightn’t have had a smear for a long period or actually might be feeling quite mortified because she’s overweight and when she’s overweight maybe her pelvic floor is… because she’s had lots of babies her pelvic floor has reduced tone so she might be having just a bit of a prolapse…But it’s just how it’s handled and the sensitivities around it because it’s a vulnerable time for some ladies.”*  *(DS300014, AS, 53 yrs)*  *“I normally take two Nurofen, to take the edge off the uncomfortable aspect of it.”*  *(DS300034, AS, 51 yrs)* |
